# Detection of phosphorothioated (PS) oligonucleotides in horse plasma using a product ion (*m/z* 94.9362) derived from the PS moiety for doping control

**DOI:** 10.1186/s13104-018-3885-5

**Published:** 2018-10-29

**Authors:** Teruaki Tozaki, Kaoru Karasawa, Yohei Minamijima, Hideaki Ishii, Mio Kikuchi, Hironaga Kakoi, Kei-ichi Hirota, Kanichi Kusano, Shun-ichi Nagata

**Affiliations:** 1 Genetic Analysis Department, Laboratory of Racing Chemistry, 1731-2 Tsurutamachi, Utsunomiya, Tochigi 320-0851 Japan; 2 AB Sciex, 4-7-35 Kitashinagawa, Shinagawa-ku, Tokyo, 140-0001 Japan; 3 Drug Analysis Department, Laboratory of Racing Chemistry, 1731-2 Tsurutamachi, Utsunomiya, Tochigi 320-0851 Japan; 4 Racehorse Hospital Ritto Training Center, Japan Racing Association, 1028 Misono, Ritto, Shiga 520-3085 Japan

**Keywords:** Gene doping, Mass spectrometry, Therapeutic nucleotides, Thoroughbred, LC/MS/MS, Phosphorothioated oligonucleotides (PSOs)

## Abstract

**Objective:**

Clinical research on gene therapy has advanced the field of veterinary medicine, and gene doping, which is the illegal use of gene therapy, has become a major concern in horseracing. Since the International Federation of Horseracing Authorities defined the administration of oligonucleotides and its analogues as a genetic therapy in 2017, the development of therapeutic nucleotide-detection techniques has become an urgent need. Most currently marketed and developed oligonucleotide therapeutics for humans consist of modified nucleotides to increase stability, and phosphorothioate (PS) modification is common.

**Results:**

We demonstrated the specific detection of phosphorothioated oligonucleotides (PSOs) using LC/MS/MS. PSOs produce the specific product ion (*m/z* 94.9362) derived from PS moiety. PS is not derived from endogenous substances in animal body, and the product ion is a suitable marker for the detection of PSOs. With our strategy, reproducible target analyses were achieved for identifying the specific substances, with a LOD of 0.1 ng/mL and a quantification rage of 0.1–200 ng/mL in deproteinated plasma. Non-target analyses could also detect the presence of PSOs selectively with 100 ng/mL in the same matrix. These results suggested that the detection of PSOs in horse blood is possible by targeting the product ion using LC/MS/MS.

**Electronic supplementary material:**

The online version of this article (10.1186/s13104-018-3885-5) contains supplementary material, which is available to authorized users.

## Introduction

Horseracing originated in the early 18th century in Britain with the production of Thoroughbreds by breeding Arabian stallions with British native mares, and it then spread to the rest of the world as a sport [1]. Horseracing involves several phases of breeding, racing, and wagering, and requires careful administration to ensure fair practices.

One major threat to the integrity of horseracing is doping [2]. Doping is generally recognized as the use of banned performance-enhancing drugs in human sports, while the use of low-molecular-weight compounds such as non-steroidal anti-inflammatory drugs (NSAIDs) in addition to anabolic steroids and proteins such as recombinant erythropoietin is also regulated and prohibited in fair horseracing [3–5].

The rules regarding the use of prohibited substances should be unified across the participants to ensure a fair race. The International Agreement on Breeding, Racing and Wagering (IABRW) was published to harmonize the rules of horseracing in member countries by the International Federation of Horseracing Authorities (IFHA) (http://www.horseracingintfed.com). In 2016, the IFHA established the Gene Doping Control Subcommittee (GDCS) to clearly define “gene doping,” “gene therapy,” “cellular therapy,” and “cellular doping.”

The IFHA defined “the administration of oligomers or polymers of nucleic acid and nucleic acid analogues” as a genetic therapy in racehorses. The oligomers should be recognized as therapeutic oligonucleotides [6], which include antisense oligonucleotides (ASOs), siRNA oligonucleotides, anti-miRNA oligonucleotides (AMOs), aptamer oligonucleotides, and CpG oligonucleotides [7–12].

In recent years, pharmaceutical industries have focused on developing new concept medicines, including therapeutic oligonucleotides. Oligonucleotides synthesized using natural nucleic acids, DNA and RNA, are degraded rapidly in the bloodstream [13]. For therapeutic efficiency, the structural integrity of the oligonucleotide drugs should be maintained for a longer period of time, which is a key process in the development of therapeutic oligonucleotides. One effective technique for enhancing stability in synthetic oligonucleotides is phosphorothioation of natural nucleic acids [14]. Currently, phosphorothioated oligonucleotides (PSOs) are being frequently used in the development of therapeutic oligonucleotides.

**Fig. 1:**
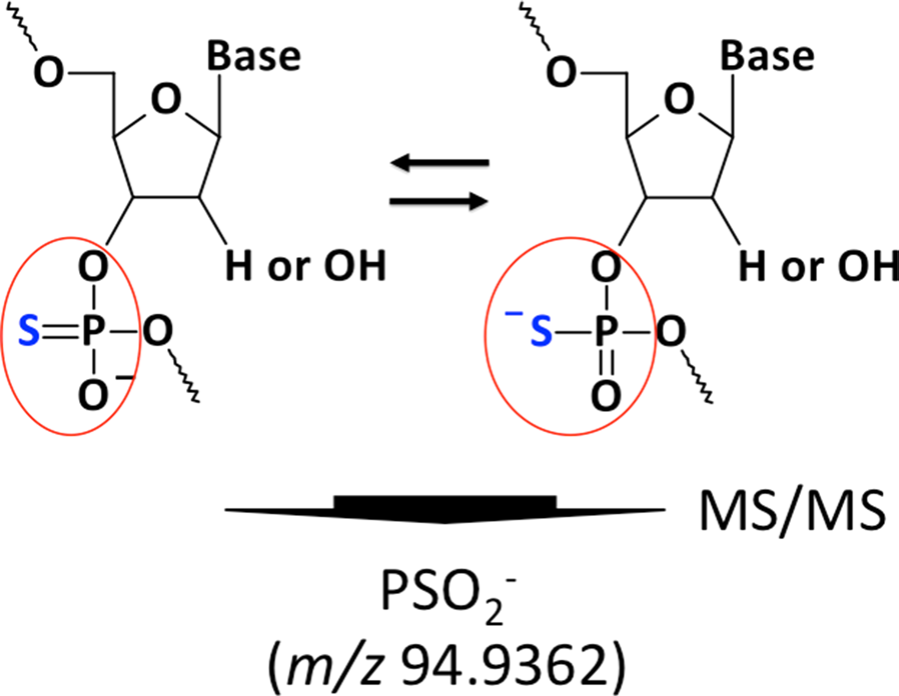
Structure of the product ion (*m/z* 94.9362) derived from phosphorothioated oligonucleotides (PSOs)

While several monitoring methods were developed for oligonucleotides [15, 16], analytical methods for doping detection are not yet well established. In this study, we focused on the phosphorothioate (PS) moiety as a marker for gene doping detection in horseracing (Fig. 1). For this, we developed and evaluated the detection method for PSOs in horse plasma by monitoring the specific product ion of anionized PS using a quadrupole-time-of-flight (Q-TOF) tandem mass spectrometer.

## Main text

### Materials and methods

#### Preparation of horse plasma

We used horse plasma, which is an ideal matrix for gene doping tests, in this study. Blood was collected in EDTA blood collection tubes, and plasma was separated by centrifugation. In 50 mL tubes, 30 mL acetonitrile (LC/MS grade, Wako, Osaka, Japan) was added to 15 mL horse plasma. The solution was mixed vigorously and stored at 4 °C for 15 min. It was then centrifuged at 3000×*g* for 15 min, and 40 mL of the supernatant was collected. After removing the solvent using an evaporator, the final volume was adjusted to 10 mL with Milli-Q water. The deproteinated plasma samples were stored at − 20 °C until use.

#### Preparation of phosphorothioated oligonucleotides (PSOs) and sample preparation for analyses

Phosphorothioated oligonucleotides was used as the model therapeutic oligonucleotide in this study. The PSO of the antisense strand of exon 2 of the horse *myostatin* gene (*MSTN*), 5ʹ-GAGATCGGATTCCAGTATACCA-3ʹ (22 mer), was synthesized (GeneDesign, Inc. Osaka, Japan) and was named oligo 1 in this study. All nucleotides in the oligonucleotide were phosphorothioated (average mass: 7080.8, monoisotopic mass: 7075.692). The PSO was purified by high-performance liquid chromatography (HPLC). Oligo dT, 5ʹ-TTTTTTTTTTTTTTT-3ʹ (15 mer), which was not phosphorothioated, was also synthesized (GeneDesign, Inc.). The oligos were dissolved in 100 mmol/L triethylamine acetate (TEAA) buffer at a concentration of 20 μg/mL. The oligos were diluted and spiked into the deproteinated plasma with an internal standard and were used as the analytical samples in this study.

#### Conditions for LC

The samples were analyzed on a Prominence UFLC XR HPLC system (Shimadzu, Kyoto, Japan) using an ACQUITY UPLC BEH C_18_ column (1.7 μm, 2.1 × 30 mm, Waters, Milford, MA, U.S.A.), which was maintained at 60 °C. The mobile phase consisted of solvent A (100 mM hexafluoro-2-propanol [HFIP] and 10 mM triethylamine [TEA]) and solvent B (methanol), with gradient elution as follows: 5 to 50 to 90% B in 2 and 0.01 min and hold for 0.49 min, then back to 5% B in 0.01 min, and hold for 1.49 min after pre-equilibrium in 5% solvent B for 0.5 min at a flow rate of 0.4 mL/min. Sample injection volume was 2 μL.

#### Conditions for MS

A quadrupole-time-of-flight (Q-TOF) tandem mass spectrometer equipped with a DuoSpray™ ion source (TripleTOF^®^ 6600 System, SCIEX, Framingham, MA, U.S.A.) was used.

For target analyses, a product ion scan was carried out using the precursor ion of *m/z* 785.18 corresponding to [M−9H]^9−^, and the Q1 resolution was set to low to transmit the isotope ions and increase the sensitivity. Then, the product ion of *m/z* 94.9362 derived from the PS moiety was monitored using High-Resolution Multiple Reaction Monitoring (MRM^HR^) in high sensitive mode and enhance ion mode. The MS parameters for the scan range, gas 1 (nebulizer gas), gas 2 (heater gas), curtain gas, temperature, ion spray voltage, declustering potential, and collision energy (CE) were set as follows: *m/z* 70–300, 60 psi, 80 psi, 25 psi, 600 °C, − 4000 V, − 120 V, and − 92 V, respectively. Mass spectrometer was calibrated with external standard solution before acquisition.

For non-target analyses, Data Dependent Acquisition (DDA) was used to search for the PSOs that could produce the specific product ion of negatively charged PS. It was possible to acquire product ion scan data by triggering the 10 most intense precursor ions within the DDA criteria every 0.2 s cycle. The MS parameters were the same as in the target analyses except for the scan ranges: *m/z* 450–2800 for full scan and *m/z* 70–2800 for product ion scan, respectively.

Analyst^®^TF 1.7.1 Software (SCIEX), MultiQuant™ 3.0.2 Software (SCIEX), and PeakView^®^ 2.2 Software (SCIEX) were used for data acquisition, target analysis, and non-target analysis, respectively. The peak areas of analyte vs. nominal analyte concentrations were fitted by quadratic regression using 1/x^2^ weighting factors.

### Results

#### Method development and optimization

Optimization for LC conditions (column, eluent, and gradient condition) was performed based on the peak height, peak shape, and signal to noise ratio. MS conditions for oligo 1 (PSO) were examined by flow injection analysis under the LC conditions described above. Multiply charged ions with charge numbers of 3–10 were detected in negative ion mode, and the most-intense-charged ion ([M−9H]^9−^, theoretical *m/z* 785.18) was selected as a precursor ion for target analysis (Additional file 1: Figure S1a). The specific and most abundant product ion of *m/z* 94.9362 derived from the PS moiety was observed when CE was set for − 92 V (Additional file 1: Figure S1b).

In addition, the peak intensity of *m/z* 94.9362 on MS/MS was dramatically improved by approximately 16 times in enhance mode, which enabled the targeted product ions to reach the detector more efficiently (Additional file 1: Figure S1c, d). Therefore, target analyses in MRM^HR^ were conducted using the transition of *m/z* 785.18–94.9362 using a 20 mDa window. Other parameters were set so that the maximum detection of the target compound in MRM^HR^ was obtained.

#### Target analysis

Based on the results of the analysis of spiked plasma, the target analyte in deproteinated plasma could be detected with a limit of detection (LOD) of 0.1 ng/mL. We also evaluated the quantitative performance using curve fitting, and reproducibility, assuming that quantitative analysis would be required for pharmacokinetic studies in drug development. For quantitative analyses, good curve fittings were obtained in the range of 0.1–200 ng/mL with a correlation coefficient r ≥ 0.990 and 82–118% accuracy, and within-day and day-to-day reproducibilities were confirmed (Additional file 1: Figure S2, Table S1).

#### Non-target analysis

Non-target analysis was conducted using DDA to determine whether a sample contained any PSOs in endogenous components. The specific product ion of *m/z* 94.9362 can be from PSOs, but not from endogenous components.

**Fig. 2:**
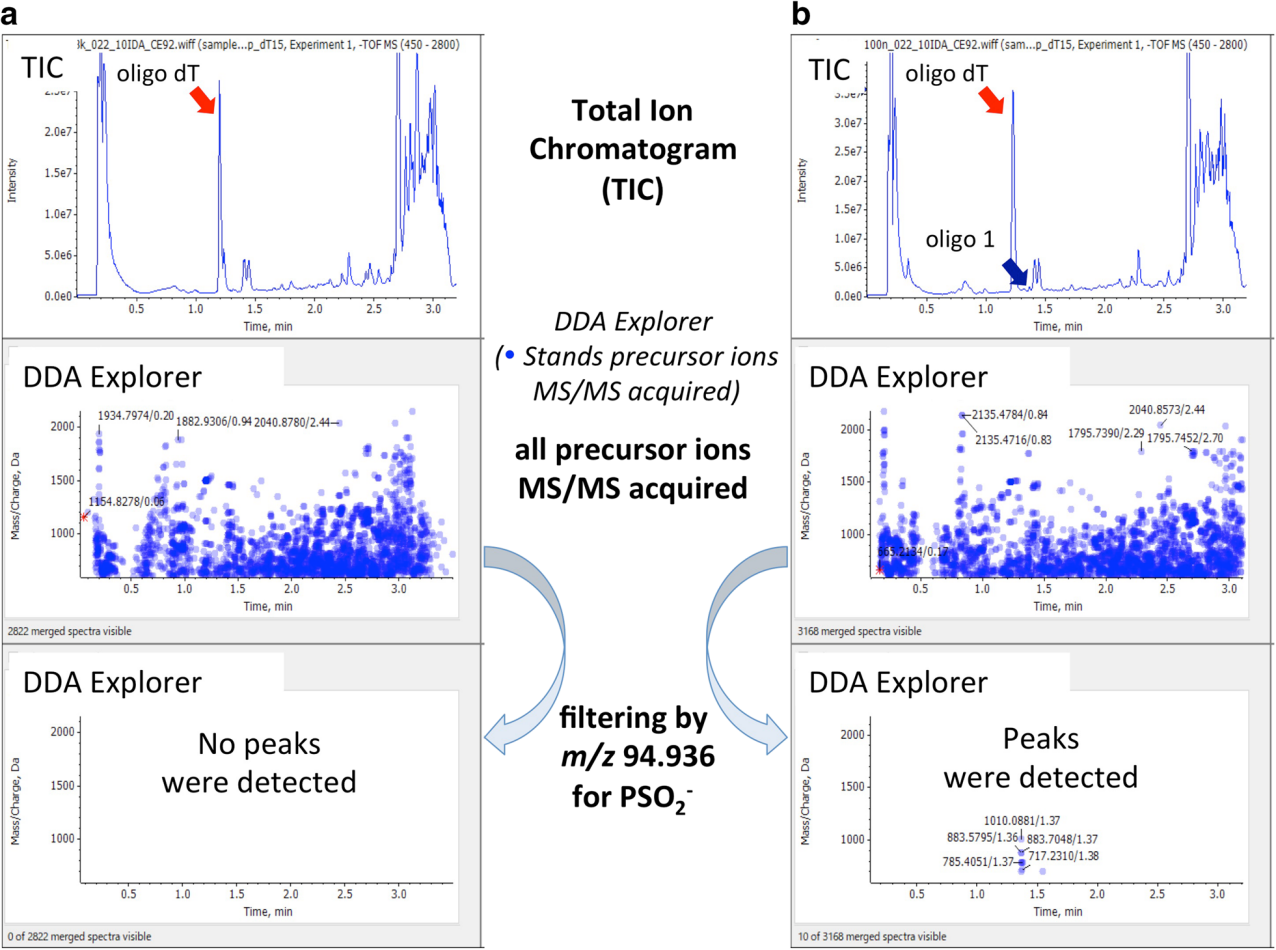
Non-target analyses of phosphorothioated oligonucleotides (PSOs) in deproteined plasma. **a** Total ion chromatogram (TIC), data dependent acquisition (DDA) Explorer (Time versus Precursor MS/Charge for DDA dependents), DDA Explorer filtered by *m/z* 94.936 with 10 ppm mass tolerance of deproteinated plasma with 4.5 µg/mL oligo dT. **b** TIC, DDA Explorer, and DDA Explorer filtered by *m/z* 94.936 with 10 ppm mass tolerance of 100 ng/mL oligo 1 in deproteinated plasma with 4.5 µg/mL oligo dT. Phosphorothioated (PS) oligomers were selectively detected in a heavy biological matrix containing a 45 times higher concentration of a non-PS oligomer without any sequence information

The blank (matrix: deproteined plasma including 4.5 µg/mL oligo dT) and the samples spiked at concentrations of 1–20,000 ng/mL in the same matrix were analyzed with DDA using the specific product ion of *m/z* 94.9362 as model case studies (Fig. 2a, b). The spiked analyte was detected at a retention time of close to 1.4 min at concentrations of 100 ng/mL (Fig. 2b). In contrast, no interfering peaks were detected in the blank sample (Fig. 2a). In addition, peaks derived from oligo dT (non-modified oligonucleotide) were not detected.

### Discussion

Synthetic oligonucleotides containing natural nucleic acids, such as DNA and RNA, are quickly degraded by nucleases when administered into the body [13]. Therefore, almost all therapeutic oligonucleotides are modified to enhance structure stability, and phosphorothioation is one of the most common structural modifications [14]. PS compounds do not naturally occur in animals (humans and horses), and they can be clearly distinguished from endogenous components in plasma by monitoring the specific product ions of PS. Therefore, we focused on the detection of the PS moiety as a method for identifying gene doping in racehorses.

For target analysis, we successfully detected PSO in deproteinated horse plasma with an LOD of 0.1 ng/mL. Furthermore, good curve fittings and reproducibility were confirmed in the range of 0.1 to 200 ng/mL, indicating that the product ion (*m/z* 94.9362) would be a good marker to detect and monitor PSOs in horse blood for gene doping and medicine development.

Because the sequences of therapeutic oligonucleotides are species specific, it is unlikely that medicines developed for humans would be used for horses. However, it is easy to design antisense oligonucleotides for therapeutic nucleotides based on horse genomic information in the public databases [17]. In addition, oligonucleotides containing nucleic acids or nucleic acid analogs can be readily purchased. Therefore, non-certified oligonucleotides rather than approved oligonucleotides would be used for doping in the racing industry. This suggests that the development of detection techniques based on non-target analyses is required for doping control of oligonucleotides in racehorses.

For non-target analysis, exhaustive scanning of PSOs in deproteinated plasma using the DDA function revealed that the product ions (*m/z* 94.9362) could be detected with 100 ng/mL. PSOs were selectively detected in the complex biological matrix, including natural nucleic acids, without any sequence information. The results suggested that DDA of a Q-TOF tandem mass spectrometer is a useful method for the detection of unknown PSOs used for doping in the same manner as the model samples used in this study.

In conclusion, the product ion (*m/z* 94.9362) is an effective marker for the detection of non-approved PSOs used for doping. The product ion is also a good marker for the pharmacokinetics of PSOs because of its quantitative sensitivity.

## Limitations

The limitation of this study is a model case report that used phosphorothioated (PS) oligonucleotides spiked in deproteinated plasma. Therefore, studying with animal models is recommended.

## Additional file

**Additional file 1: Figure S1.** Mass spectra of full scan and product ion scan for phosphorothioated oligonucleotides (PSOs). **Figure S2.** Quadratic calibration curves of phosphorothioated oligonucleotides (PSOs) in deproteined plasma. **Table S1.** Accuracy and reproducibility of phosphorothioated oligonucleotides (PSOs) detection.

## Abbreviations

AMO: anti-miRNA oligonucleotide
ASO: antisense oligonucleotide
LOD: limit of detection
MS: mass spectrometer
NSAID: non-steroidal anti-inflammatory drug
PSO: phosphorothioated oligonucleotide
Q-TOF: quadrupole-time-of-flight
