# Supplementary material for: Detection of phosphorothioated (PS) oligonucleotides in horse plasma using a product ion (m/z 94.9362) derived from the PS moiety for doping control
Source: BMC Res Notes. 2018 Oct 29;11:770. doi: 10.1186/s13104-018-3885-5 (PMC6206624; doi:10.1186/s13104-018-3885-5)
Supplement: Supplementary file 1 — Additional file 1: Figure S1. Mass spectra of full scan and product ion scan for phosphorothioated oligonucleotides (PSOs). Figure S2. Quadratic calibration curves of phosphorothioated oligonucleotides (PSOs) in deproteined plasma. Table S1. Accuracy and reproducibility of phosphorothioated oligonucleotides (PSOs) detection. [file 13104_2018_3885_MOESM1_ESM.pdf]

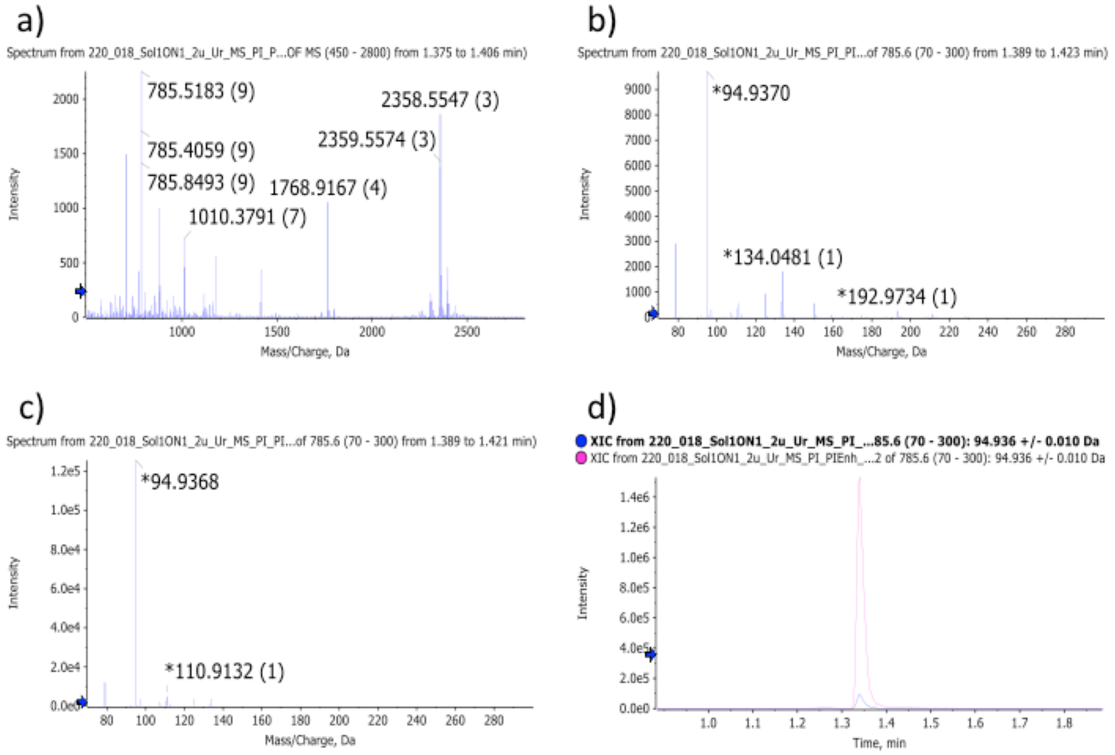

Figure S1. Mass spectra of full scan and product ion scan for phosphorothioated oligonucleotides (PSOs)

a) Mass spectra of oligo 1, b, c) Product ion spectrum of oligo 1 ($[M{-}9H]^{9-}$) without and with enhancement of *m/z* 94.9362 derived from the PS moiety, d) Overlaid extracted ion chromatogram (EIC) of *m/z* 94.9362 with and without enhancement in blue and pink, respectively.

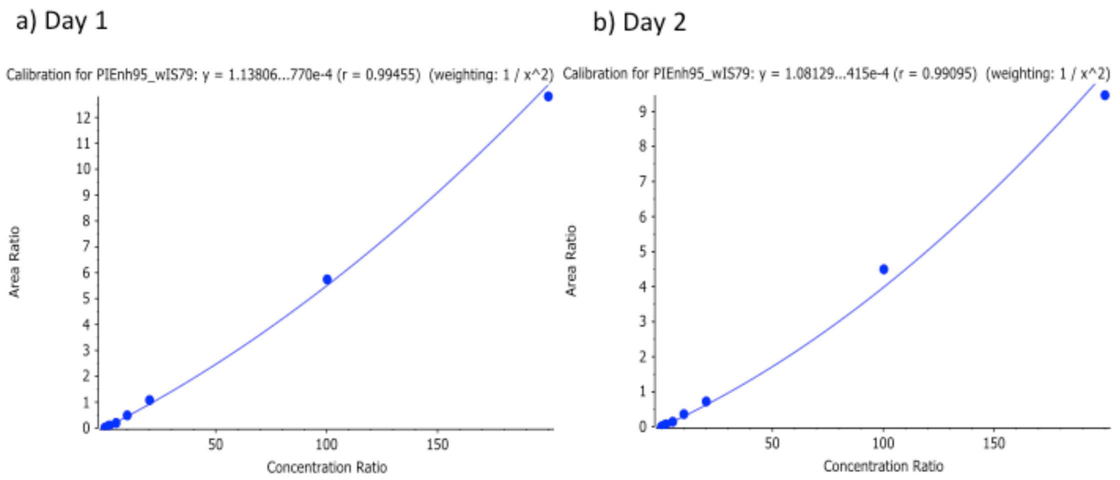

Figure S2. Quadratic calibration curves of phosphorothioated oligonucleotides (PSOs) in deproteined plasma

a) Day 1, b) Day 2.

Table S1. Accuracy and reproducibility of phosphorothioated oligonucleotides (PSOs) detection.

Spiked samples	Concentration (ng/mL)	Accuracy (%)	
		Day 1	Day 2
STD	0.1	103.0	108.5
	0.2	103.5	93.4
	0.5	82.5	82.0
	1.0	89.8	86.6
	2.0	91.6	94.2
	5.0	98.7	90.9
	10.0	113.1	117.4
	20.0	114.7	117.2
	100.0	104.0	109.8
	200.0	97.4	95.4
QC	20.0	111.5	119.3
	200.0	96.1	102.4
